# Applying the Just-In-Time Adaptive Intervention Framework to the Development of Gambling Interventions

**DOI:** 10.1007/s10899-023-10250-x

**Published:** 2023-09-02

**Authors:** Nicki A. Dowling, Simone N. Rodda, Stephanie S. Merkouris

**Affiliations:** 1 School of Psychology, Deakin University, Geelong, Australia; 2 Melbourne Graduate School of Education, University of Melbourne, Parkville, Australia; 3 Department of Psychology and Neuroscience, Auckland University of Technology, Auckland, New Zealand

**Keywords:** Mobile health, Just-in-time adaptive intervention, Ecological momentary intervention, Microrandomised trial, Gambling, Treatment

## Abstract

Just-In-Time Adaptive Interventions (JITAIs) are emerging “push” mHealth interventions that provide the right type, timing, and amount of support to address the dynamically-changing needs for each individual. Although JITAIs are well-suited to the delivery of interventions for the addictions, few are available to support gambling behaviour change. We therefore developed *GamblingLess: In-The-Moment* and *Gambling Habit Hacker*, two smartphone-delivered JITAIs that differ with respect to their target populations, theoretical underpinnings, and decision rules. We aim to describe the decisions, methods, and tools we used to design these two treatments, with a view to providing guidance to addiction researchers who wish to develop JITAIs in the future. Specifically, we describe how we applied a comprehensive, organising scientific framework to define the problem, define just-in-time in the context of the identified problem, and formulate the adaptation strategies. While JITAIs appear to be a promising design in addiction intervention science, we describe several key challenges that arose during development, particularly in relation to applying micro-randomised trials to their evaluation, and offer recommendations for future research. Issues including evaluation considerations, integrating on-demand intervention content, intervention optimisation, combining active and passive assessments, incorporating human facilitation, adding cost-effectiveness evaluations, and redevelopment as transdiagnostic interventions are discussed.

## Introduction

Mobile health (mHealth) interventions, which use mobile or wireless technologies to promote health (World Health Organization [WHO], 2017), can extend the provision of support for changing health behaviours beyond that provided by standard treatments. mHealth interventions have many advantages, such as their accessibility, availability, convenience, anonymity, transportability, and cost-effectiveness (Bakker et al., 2016; Carpenter et al., 2020; Heron and Smyth, 2010; Kim et al., 2019; Klasnja & Pratt, 2012; Walton et al., 2018). They also have a high potential as low-burden and scalable interventions that can accurately record data and be translated to the real-world (Bakker et al., 2016; Carpenter et al., 2020; Heron and Smyth, 2010; Kim et al., 2019; Klasnja & Pratt, 2012; Walton et al., 2018). This modality also offers the unique potential to meet the needs of populations that are underserved by traditional treatments, including people who are not able or willing to engage in other treatments, by reducing geographic, financial, or social help-seeking barriers (Bakker et al., 2016; Heron and Smyth, 2010; Kim et al., 2019). mHealth interventions, which most often involve health or motivational messages, reminders, or support, can supplement traditional treatments or be employed as stand-alone treatments (Heron & Smyth, 2010). They can be “pull” interventions that are initiated by individuals when they want support, or “push” interventions that are initiated by computerised intervention protocols to decide when and how support should be offered (Klasnja et al., 2015; Walton et al., 2018).

### Just-In-Time Adaptive Interventions

Just-In-Time Adaptive Interventions (JITAIs) are a suite of increasingly popular “push” mHealth intervention designs that tailor the type, timing, and amount of support toaddress each individual’s dynamically-changing needs (Carpenter et al., 2020; Nahum-Shani et al., 2015; Nahum-Shani et al., 2014; Nahum-Shani et al., 2018; Walton et al., 2018). *Just-in-time* support refers to providing the right type, timing, or amount of support, while *adaptive* refers to the use of dynamic information to repeatedly deliver this support to maximise outcomes (Collins et al., 2004; Nahum-Shani et al., 2015; Nahum-Shani et al., 2014; Nahum-Shani et al., 2018; Wang and Miller, 2020). Across various disciplines, mHealth interventions that share similar just-in-time and adaptive components have also been described as dynamic tailoring (Krebs et al., 2010), intelligent real-time therapy (Kelly et al., 2012), and individually and dynamically-tailored ecological momentary interventions (Heron & Smyth, 2010).

JITAIs aim to prevent negative health outcomes and/or promote positive health behaviours (Klasnja et al., 2015; Nahum-Shani et al., 2014). They are developed to provide support when people are: (a) susceptible to negative health outcomes (*states of vulnerability*) and/or positive health behaviour change (*states of opportunity*); and (b) able and/or willing to receive and employ the support (*states of receptivity*) (Nahum-Shani et al., 2015; Nahum-Shani et al., 2018). In everyday settings, these states can rapidly emerge across individuals and over time within individuals (Shiffman, 2009; Shiffman et al., 2008; Stone & Shiffman, 1994). JITAIs leverage mobile or wireless technologies, including smartphone-embedded or wearable sensors and smartphone-delivered ecological momentary assessments (EMAs), to continuously monitor these dynamically-changing internal states and situational contexts in real-time to identify the type and timing of providing support, while attempting to maximise uptake and impact and minimise burden, disruption, and habituation (Carpenter et al., 2020; Klasnja et al., 2015; Nahum-Shani et al., 2015; Nahum-Shani et al., 2014; Nahum-Shani et al., 2018).

There is empirical evidence that JITAIs are effective in changing behaviour across several health domains, including healthy diet, post-traumatic stress, depression, anxiety/stress, pain, bipolar disorder, weight loss, addiction, diabetes management, and physical activity (Carpenter et al., 2020; Heron & Smyth, 2010; Nahum-Shani et al., 2014, 2018; Wang & Miller, 2020). A systematic review and meta-analysis of 33 empirical JITAI studies conducted from 2008 across health domains revealed moderate-to-large effect sizes for JITAIs relative to both waitlist-control conditions (k = 9, Hedge’s g = 1.65) and non-JITAI treatment conditions (k = 21, Hedge’s g = 0.89; Wang and Miller, 2020). JITAIs are well-suited to the delivery of interventions across the addictions, given that use episodes or lapses are precipitated by discrete but fluctuating states (e.g., motivation, urges or cravings) or events (e.g., high-risk situations; Carpenter et al., 2020; Goldstein et al., 2017; Heron and Smyth, 2010; Witkiewitz and Marlatt, 2004). Indeed, there is evidence that JITAIs are feasible, acceptable, credible, and effective in addressing smoking (Brendryen et al., 2008; Brendryen & Kraft, 2008; Businelle et al., 2016; Free et al., 2011; Hebert et al., 2020; Naughton et al., 2016; Riley et al., 2008; Rodgers et al., 2005; Vidrine et al., 2006), binge drinking (Suffoletto et al., 2012), heavy drinking (Weitzel et al., 2007), heavy drinking and smoking (Witkiewitz et al., 2014), and alcohol use disorders (Gonzalez & Dulin, 2015; Gustafson et al., 2014; Moody et al., 2018).

### Gambling Just-In-Time Adaptive Interventions

Despite growing evidence of their efficacy, only a small number of JITAIs have been developed to support gambling behaviour change. Two smartphone apps, Smartphone-based Problem Gambling Evaluation and Technology Testing Initiative (*SPGeTTI*; Humphrey et al., 2019) and *Don’t Go There* (Coral et al., 2020), employ geolocation sensors (GPS, gyroscopes, accelerometers, and magnetometers) to deliver notifications when they detect that individuals are close to land-based gambling venues. *SPGeTTI* also includes on-demand intervention content (gambling diary, self-monitoring tips for relapse prevention, and contacts for help services), while *Don’t Go There* includes a feature that enables an elected health professional to access the user’s information. Low rates of recruitment precluded a planned randomised controlled trial (RCT) evaluating *SPGeTTI*, whereby only four participants completed the study. Focus group interviews with a separately recruited sample of 20 gamblers revealed a high interest in the use of JITAIs for intervention delivery, but specific issues with the *SPGeTTI* app, including excessive battery drainage. *Don’t Go There* is currently in the development stage, with a usability study planned.

Two other smartphone apps collect dynamic information from EMAs to initiate the delivery of real-time adaptive interventions. *Jeu-contrôle*, which is a publicly available smartphone app that has not yet been subject to evaluation, employs EMAs to provide personalised feedback to support time and expenditure limit adherence (Khazaal et al., 2017). *GamblingLess: Curb Your Urge* is a smartphone app-delivered intervention based on the relapse prevention model, which aims to prevent subsequent gambling episodes by reducing gambling cravings (Hawker et al., 2021; Merkouris et al., 2020). This individually- and dynamically-tailored EMI, which was adapted from *GamblingLess*, an evidence-based online self-directed program (Dowling et al., 2018, 2021; Hawker et al., 2021; Humphrey et al., 2020; Humphrey et al., 2022; Merkouris et al., 2020; Merkouris et al., 2017; S. N. Rodda et al., 2019), tailors gambling craving management activities in response to repeated EMAs measuring craving intensity. These intervention activities are also available ‘on-demand’. Usability testing revealed that 29 key stakeholders (consumers, gambling clinicians, and gambling researchers) (Hawker et al., 2021; Merkouris et al., 2020) highly rated the intervention content, helpfulness, acceptability, and usability. In a pilot study (Hawker et al., 2021), participants demonstrated a more than 70% reduction in the average number of gambling episodes and cravings during the 4-week intervention period, as well as a 10% reduction in craving intensity immediately after a treatment activity. At the post-intervention and one-month follow-up evaluations, participants reported significant medium-to-large reductions in gambling symptom severity, gambling frequency and expenditure, cravings, and self-efficacy. In an evaluation of the clinical impact of the JITAI, just under half of all participants (48%) reported either recovery or improvement in the severity of their gambling symptoms at the follow-up evaluation.

### Review Manuscript Aims

We have recently developed two theoretically-informed and evidence-based JITAIs. The first, *GamblingLess: In-The-Moment* (Dowling et al., 2022), is part of a suite of gambling online and mHealth interventions that builds on the pilot trial data of *GamblingLess: Curb Your Urge* (Hawker et al., 2021; Merkouris et al., 2020), while the second, *Gambling Habit Hacker* (Rodda et al., 2022), forms part of a suite of implementation support interventions based on lived experience across the addictions (Brittain et al., 2021; Park et al., 2020; Rodda et al., 2020; S. N. Rodda, N. Booth, Rodda et al., 2018a, c, d). In response to a call for continued communication regarding the need to develop and evaluate JITAIs (Goldstein et al., 2017), this review manuscript aims to describe the decisions, methods, and tools we used to design these two JITAIs. This review manuscript complements the protocol papers for these JITAIs (Dowling et al., 2022; Rodda et al., 2022), which describe their initial evaluation and optimisation protocols. In contrast, this manuscript describes the steps we took to develop *GamblingLess: In-The-Moment* and *Gambling Habit Hacker* and *how and why* we made the decisions we did, with a view that sharing our approach will providing guidance and encouragement to addiction researchers who wish to develop JITAIs in the future (Goldstein et al., 2017).

We modelled this review manuscript on a similar paper authored by Goldstein et al. (2017), which describes their approach when developing a JITAI targeting lapses following a weight control diet. Like Goldstein et al. (2017), we describe how and why we made certain decisions when we applied the comprehensive, organising scientific framework developed by Nahum-Shani and colleagues (2015; 2014; 2018) to guide the JITAI design. In this framework, four components are described: (1) *decision points* (timepoints at which intervention delivery decisions are made); (2) *intervention options* (possible types, doses, timings, and delivery modes of the support that may be provided at each decision point); (3) *tailoring variables* (information relating to a person’s ecological context or internal state that is employed to identify when and/or how intervention options are delivered); and (4) *decision rules* (rules that specify which intervention option is to be offered, and when, for each individual at each level of the tailoring variables). These components are primarily guided by the distal outcome (long-term intervention goal), but also by multiple proximal outcomes (short-term intervention goals) (Nahum-Shani et al., 2018). Nahum-Shani et al. (2015) organises these components into three areas: (1) defining the problem, (2) defining what just-in-time means in the context of the problem, and (3) formulating the adaptation strategy. We found Goldstein et al.’s (2017) application of this framework to a specific JITAI incredibly helpful in informing our decision-making across the development and evaluation phases for our JITAIs; and we offer this manuscript in the same spirit.

Our review manuscript first provides an overview of each of our JITAIs, followed by why we selected the behaviour change theories that guided their construction and how we applied the scientific framework to guide their design. We conclude the manuscript with a discussion of how and why we applied micro-randomised trials (MRTs) to enable the optimisation of these JITAIs, followed by the key logistical and methodological challenges we faced during the development and evaluation phases, as well as considerations for future research.

## Overview of the JITAIs

In line with recommendations (Nahum-Shani et al., 2018), the development of both *GamblingLess: In-The-Moment* and *Gambling Habit Hacker* involved a multidisciplinary collaboration with expertise drawn from clinical psychology, social psychology, biostatistics, research design, implementation science, and technology development. Both apps will be subject to 28-day MRTs, accompanied by within-group follow-up evaluations across a six-month period and acceptability evaluations (Dowling et al., 2022; Rodda et al., 2022). Both apps are available for download during the trial period on Android (Google Play Store) and Apple (App Store) devices.

### GamblingLess: In-The-Moment

**Fig. 1:**
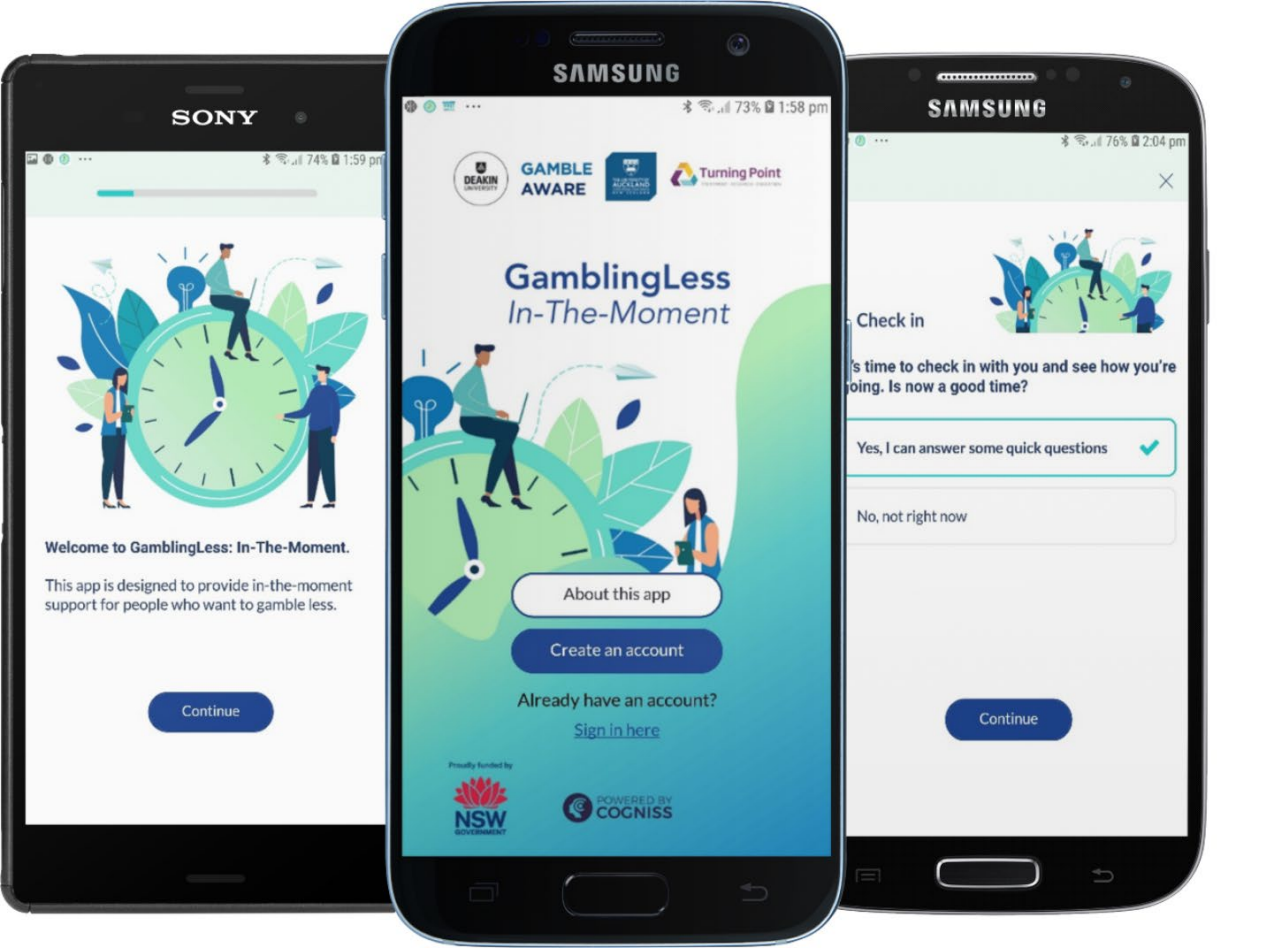
Illustrative screenshots of *GamblingLess: In-The-Moment*: Welcome page, create an account, check-in (EMA) “snooze”

*GamblingLess: In-The-Moment* is one digital offering in a suite of gambling mHealth interventions that are evidence-based and theoretically-informed. It is a smartphone app-delivered JITAI for people who want to quit or reduce their gambling. The aim of this JITAI is to provide the type and amount of support required at times when people are cognitively vulnerable (i.e., when they report high-intensity cravings, low self-efficacy, or positive gambling outcome expectancies) to reduce the likelihood of a subsequent gambling episode. The long-term goal is to reduce gambling symptom severity (*distal outcome*) via the short-term goal of reducing the likelihood of gambling episodes (*primary proximal outcome*). This reduction in the probability of gambling episodes is posited to be achieved through reductions in craving intensity, improvements in self-efficacy, or reductions in positive outcome expectancies (*secondary proximal outcomes*). In this JITAI, we created *decision rules* that specify that individuals who are available for treatment (i.e., in a state of receptivity) and report a state of cognitive vulnerability (characterised by high craving intensity, lowered self-efficacy, and high positive outcome expectancies: *tailoring variables*) in EMAs delivered at three semi-random times each day (*decision points*) are delivered tailored cognitive-behavioural and third-wave intervention activities designed to address these cognitive processes (*intervention options*). The JITAI is designed to be used as a standalone or adjunctive treatment when individuals are actively gambling or to prevent relapse during recovery. Illustrative screenshots of *GamblingLess: In-The-Moment* are displayed in Fig. 1.

### Gambling Habit Hacker

**Fig. 2:**
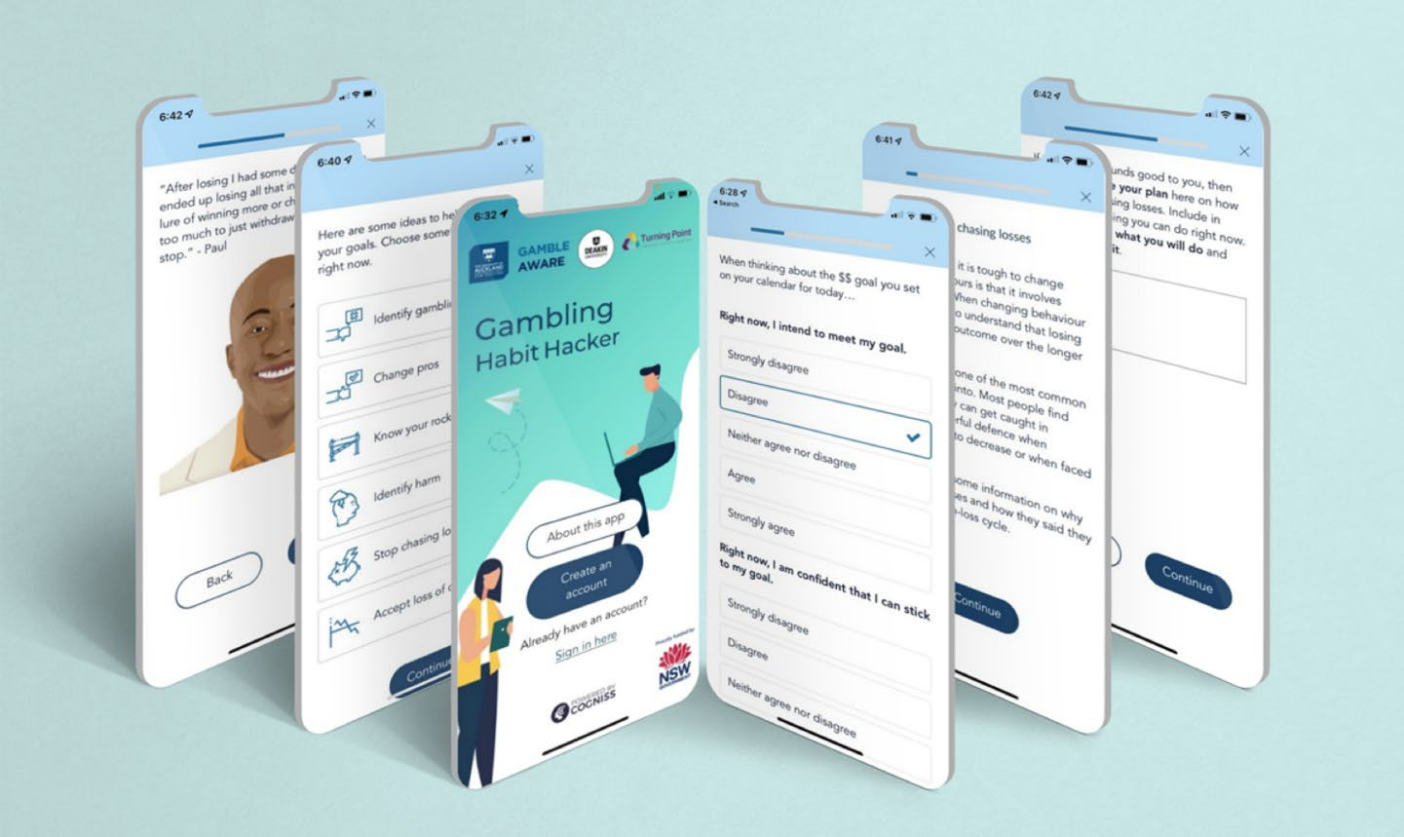
Illustrative screenshots of *Gambling Habit Hacker* app: Lived experience quote; individual strategy menu; create an account; check-in (EMA); individual strategy page; action planning

*Gambling Habit Hacker* is a digital offering in a suite of treatments for addictive behaviours delivering implementation support that has been developed using lived experience research. It is a smartphone app-delivered JITAI for people who want to improve their ability to adhere to their gambling expenditure limits (i.e., goals). This JITAI aims to provide the type of support required at times of goal vulnerability (low strength of intention [to adhere to their gambling expenditure limits], low goal self-efficacy, low urge self-efficacy, and high-risk situations) to enhance adherence to gambling expenditure limits. The long-term goal is to reduce gambling expenditure (*distal outcome*) via the short-term goal of increased adherence to gambling expenditure limits (*primary proximal outcome*). This increased adherence to gambling expenditure limits is posited to be achieved via increased strength of intention, increased goal self-efficacy, and increased urge self-efficacy (*secondary proximal outcomes*). In this JITAI, we created decision rules that specify that participants who are available for treatment (i.e., in a state of receptivity) and indicate that they are in a state of goal vulnerability (characterised by low strength of goal intention, low goal self-efficacy, low urge self-efficacy, or a high-risk situation: *tailoring variables*) in EMAs sent at three semi-random times a day (*decision points*) are encouraged to engage in action and coping planning activities designed to facilitate the use of behaviour change strategies (*intervention options*). Although individuals undertake the planning activities within the app, the implementation of the plan is conducted in the real world. This JITAI is intended for use as a standalone intervention across the entire period of post-intentional action, inclusive of longer term maintenance of behaviour change. Illustrative screenshots of *Gambling Habit Hacker* are displayed in Fig. 2.

## Theories of Behaviour Change

There is consensus in the literature that existing behaviour change theories are limited in their ability to guide the construction of JITAIs as they fail to describe the dynamic processes underlying states for receptivity, vulnerability, and opportunity (Nahum-Shani et al., 2015; Nahum-Shani et al., 2014; Nahum-Shani et al., 2018; Riley et al., 2011; Spruijt-Metz and Nilsen, 2014). Theories that explain the emergence of a state of vulnerability and/or opportunity as a dynamic process involving the interaction of stable and transient factors are most helpful, but still lack specification about the temporal relationships between factors in a manner that informs the type, timing, and amount of support provided (Klasnja et al., 2015; Nahum-Shani et al., 2015; Nahum-Shani et al., 2014; Nahum-Shani et al., 2018).

### GamblingLess: In-The-Moment

In the absence of such refined behaviour change theories, we employed the reformulated relapse prevention model (Witkiewitz & Marlatt, 2004) as the guiding theoretical framework for the development of *GamblingLess: In-The-Moment*. This model explains the likelihood of relapse resulting from the multidimensional, non-linear, and dynamic interactions between various antecedents within high-risk situations. These antecedents include background factors (e.g., family history, comorbid psychopathology, years of dependence, social support), physiological states (e.g., physical withdrawal), cognitive processes (e.g., craving, self-efficacy, positive outcome expectancies, motivation, and the abstinence violation effect), affective states, and coping skills. In this model, responses to high-risk situations are related to distal and proximal precipitants, which operate within tonic processes and phasic responses. Tonic processes, which are distal risks or stable background factors that determine *who* is vulnerable for relapse, tend to accumulate to set the foundation for the possibility of relapse. They set the initial threshold for relapse and often lead to the initiation of a high-risk situation. In contrast, phasic responses, which are proximal or situational processes fluctuating over time and contexts, determine *when* relapse will occur. Momentary coping responses, however, can also be considered to be phasic events that influence the degree to which a high-risk situation will result in a lapse. Feedback loops are included in the model, whereby lapses may reciprocally impact on the same factors (i.e., cognitive processes, affective states and coping behaviour) that contributed to them. The reformulated relapse prevention model has received substantial empirical support across a range of addictive behaviours (Menon & Kandasamy, 2018; Witkiewitz and Marlatt, 2004).

In this model, cognitive processes are conceptualised as both tonic processes and phasic responses, whereby relatively stable cognitive processes, such as global self-efficacy and outcome expectancies, may serve to function as tonic processes; while cognitive processes that fluctuate over time and contexts, such as cravings or momentary changes in self-efficacy and outcome expectancies, may serve to function as phasic responses. Considerable cross-sectional evidence suggests that cravings, self-efficacy, and positive outcome expectancies, are associated with problem gambling severity, gambling abstinence, and gambling relapse; and that these cognitive processes improve following face-to-face and self-directed interventions (See Dowling et al., 2022 for a review of this literature). Although there is less empirical evidence relating to the degree to which these cognitive processes act as phasic responses, several EMA studies (Dowling et al., 2020; Hawker et al., 2020) suggest that cravings and transient changes in self-efficacy, but not transient changes in positive outcome expectancies, are associated with the likelihood of subsequent gambling episodes in real-time. Moreover, these studies suggest that all of these cognitive processes interact in real-time with other factors explicated by the relapse prevention model, such as self-efficacy, coping motives, cravings, high-risk positive reinforcement situations, positive emotional states, and coping styles. These findings suggest that these momentary cognitive processes are potential intervention targets and mechanisms of change for JITAIs aiming to reduce gambling behaviour. Accordingly, many of the JITAIs employed in addiction science have successfully delivered intervention content tailored to contextual features highlighted by the relapse prevention model to prevent episodes or lapses (Carpenter et al., 2020).

### Gambling Habit Hacker

The guiding theory for *Gambling Habit Hacker* is the Health Action Planning Approach (HAPA) (Schwarzer & Luszczynska, 2008), with delivery of intervention content aligned with Self-Determination Theory (Deci & Ryan, 2008; Ntoumanis et al., 2021; Sheeran et al., 2020). The HAPA is a social cognitive model that aims to address the theorised gap between intention and behaviour (Schwarzer, 2008; Schwarzer and Luszczynska, 2008; Sutton, 2008). According to the model, behaviour change involves a continuous 2-phase process involving motivation and volition, whereby an individual regularly sets and reviews priorities or goals and makes decisions on whether action is important. The motivation phase involves intention formation through the realisation that behaviour needs to change, a belief that change is worthwhile, prioritisation of change over other competing demands, and a belief that the selected action can be implemented by the individual (task self-efficacy). The volitional phase involves movement towards implementing behaviour change intentions with specific implementation planning techniques, such as action planning and coping planning. Action planning determines when, where, and how actions are taken (Sniehotta et al., 2005). In contrast, coping planning specifically addresses obstacles or barriers to implementing the action (Gollwitzer, 1999; Sniehotta et al., 2005). Operationalised as if/then planning, coping planning is used to link specific situations or events that may be barriers to implementing the action (*if*) with a specific plan that could be implemented to overcome the barrier (*then*). The purpose of advance planning is to prepare the individual to respond to barriers automatically with reduced cognitive burden in-the-moment. In the volitional phase, belief in one’s ability to maintain plans and cope with barriers that may arise (maintenance self-efficacy) and one’s ability to regain control after a failure to cope with barriers to action plan implementation (recovery self-efficacy) influence the implementation of intentions. Theoretically, both types of planning occur post-intention and prior to action but it has been suggested that coping planning is more relevant following action planning (Sniehotta et al., 2005). Meta-analytic evidence indicates that action and coping planning are effective in reducing addictive behaviours, such as smoking and alcohol use (Malaguti et al., 2020; McWilliams et al., 2019). There is also emerging evidence that gamblers can easily develop action plans but that implementation barriers can reduce the success of these plans (Rodda et al., 2020).

Finally, the overarching framework for delivery of *Gambling Habit Hacker* is Self-Determination Theory (Deci & Ryan, 2008; Ntoumanis et al., 2021; Sheeran et al., 2020), which highlights three components of motivation: autonomy, competence, and relatedness. *Gambling Habit Hacker* aims to build competence through the identification of barriers that could threaten action plans and goal adherence. We also made the decision to include prompts to develop personalised goal setting and planning to enhance autonomy and include real-world stories about the use of behaviour change strategies to foster relatedness.

## Defining the Problem

### Target Populations and Distal Outcomes

The first step in designing a JITAI is to identify a target population and a distal outcome, defined as the distal goal of the intervention, which is usually a primary clinical outcome (Carpenter et al., 2020; Nahum-Shani et al., 2015; Nahum-Shani et al., 2014; Nahum-Shani et al., 2018). Our two JITAIs have different target populations and distal outcomes to accommodate users across the continuum of gambling risk who may be experiencing harm from their gambling. As recommended (Bakker et al., 2016), we wanted to capitalise on the high accessibility of mHealth interventions to not only attract people with gambling problems, but also target sub-clinical or at-risk gamblers, particularly because these gamblers account for the majority of population-level harm due to their higher prevalence (Browne et al., 2017) and typically do not use treatment services (Bijker et al., 2022).

*GamblingLess: In-The-Moment*’s target population is people who want to reduce or quit gambling. We anticipate that this app may attract relatively high-risk gamblers but we also wanted lower-risk gamblers to benefit from the intervention. In the relapse prevention model, the distal outcome is relapse (i.e., a return to the previous problematic behaviour pattern) (Witkiewitz & Marlatt, 2004), which we operationalised as the severity of gambling symptoms.

*Gambling Habit Hacker’s* target population is people with lower severity gambling problems who want to enhance their adherence to their gambling expenditure limits. In the HAPA model, the distal outcome is a reduction in the intention-behaviour gap (Schwarzer, 2008; Schwarzer and Luszczynska, 2008; Sutton, 2008), which we operationalised as adherence to gambling expenditure limits (i.e., actual gambling expenditure relative to planned gambling expenditure). However, because we deemed it unfeasible to accurately collect daily planned gambling expenditure over long periods of time, we pragmatically selected gambling expenditure as the distal outcome. Both gambling expenditure and adherence to gambling expenditure limits therefore guided the development of the remaining JITAI components.

### Proximal Outcomes

Proximal outcomes are defined as short-term treatment goals and are evaluated straight after the treatment is provided, with a view to evaluating the efficacy of the treatment (Carpenter et al., 2020; Nahum-Shani et al., 2015; Nahum-Shani et al., 2014; Nahum-Shani et al., 2018). Defining proximal outcomes can enhance the identification of appropriate decision points, tailoring variables, decision rules, and intervention options (Nahum-Shani et al., 2018). They can be: (a) intermediate versions of the distal outcome; (b) mediators of the distal outcome (i.e., critical components in the pathways through which it is hypothesised that the intervention influences the distal outcome); and/or (c) outcomes relating to treatment engagement (defined as motivational commitment/investment in the treatment process) or intervention fatigue (defined as emotional or cognitive weariness associated with intervention engagement) (Klasnja et al., 2015; Nahum-Shani et al., 2015; Nahum-Shani et al., 2014; Nahum-Shani et al., 2018).

Consistent with the reformulated relapse prevention model, we selected gambling lapses as the primary proximal outcome for *GamblingLess: In-The-Moment*, but operationalised this intermediate version of the distal outcome as gambling episodes to reduce bias and subjectivity in reporting (Goldstein et al., 2017). Moreover, we wanted to offer gamblers the choice to select non-abstinence treatment goals, consistent with a harm minimisation approach (Dowling & Smith, 2007; Dowling et al., 2009; Ladouceur, 2005). To this end, should data allow, we may also explore whether the delivery of the intervention reduces the probability of subsequent unplanned gambling episodes. We therefore employed a 30-day Timeline Follow-Forward (an adaptation of the Timeline Follow-Back; Weinstock et al., 2004) at pre-treatment to measure planned gambling behaviour. The primary analyses, however, will explore the effect of the treatment on the probability of any subsequent gambling episode (Dowling et al., 2022). Should data allow, we may also use the Timeline Follow-Forward data to explore whether the delivery of the intervention reduces the probability of subsequent planned and unplanned gambling expenditure, which is a related variable that is not formally articulated by the relapse prevention model. Finally, although gambling episodes best represent short-term progress towards reduced gambling symptom severity, they are theoretically (Witkiewitz & Marlatt, 2004) and empirically (Dowling et al., 2020; Hawker et al., 2020) associated with specific precipitating cognitive processes (cravings, self-efficacy, and positive outcome expectancies) that could serve as tailoring variables. We therefore selected these three mediating cognitive processes as secondary proximal outcomes of reduced gambling symptom severity.

We selected adherence to gambling expenditure limits as the primary proximal outcome for *Gambling Habit Hacker*, whereby any gambling expenditure limit can be selected to accommodate both abstinence and non-abstinence treatment goals. We operationalised goal adherence as being no higher than 10% more than the planned gambling expenditure, measured using the Timeline Follow-Forward. We may also explore the influence of altering the percentage of adherence as the primary proximal outcome (e.g., 20% flexibility) or using continuous measures of adherence. This approach is consistent with the HAPA model in that it prompts individuals to form a clear intention prior to engaging in the period of behaviour change. Because it also takes into account goal vulnerability (low strength of goal intention, low goal self-efficacy, and low urge self-efficacy), we selected these three mediating processes, which could serve as tailoring variables, as proximal outcomes of goal adherence.

## Defining Just-In-Time in the Context of the Identified Problem

### Decision Points

Decision points are the points in time when a treatment decision is made (Carpenter et al., 2020; Nahum-Shani et al., 2015; Nahum-Shani et al., 2014; Nahum-Shani et al., 2018). The identification of factors that signal states of vulnerability or opportunity for proximal outcomes can help to select decision points (Nahum-Shani et al., 2015). Decision points can be made at pre-specified time intervals, at specific times of the day, at specific days of the week, or following random prompts for self-report data (Nahum-Shani et al., 2014, 2018).

The extant theoretical and empirical evidence does not provide much insight into how we should expect our proximal outcomes to be temporally related over time. The frequency with which EMAs are delivered in previous JITAIs vary considerably, ranging from once per week up to five times per day (Heron & Smyth, 2010). Hence, we considered whether we expected the process leading to our distal outcomes (reduced gambling symptom severity in *GamblingLess: In-The-Moment* and adherence to gambling expenditure limits in *Gambling Habit Hacker*), would develop over hours, days, weeks, months, or years (Nahum-Shani et al., 2015). For *GamblingLess: In-The-Moment*, momentary changes in cognitive processes (craving intensity, self-efficacy, and positive outcome expectancies) can reasonably be expected to occur at any given minute, thereby potentially leading to immediate reactivity in the form of a gambling episode. Similarly, for *Gambling Habit Hacker*, strength of intention, goal and urge self-efficacy, and whether an individual is in an internal or situational high-risk situation, can change quickly, thereby increasing risk for non-adherence to gambling expenditure limits. However, decision points at every minute require frequent assessments of these states of vulnerability to avoid missed opportunities for intervention provision. Moreover, our primary proximal outcomes (gambling episodes and non-adherence to expenditure limits) occur less frequently (Dowling et al., 2020; Hawker et al., 2020), suggesting that a less intensive EMA protocol may be required (Heron & Smyth, 2010; Kim et al., 2019).

We therefore selected three decision points at random times during three pre-specified periods each day: 8:30am-11:00am (morning), 1:00pm-3:30pm (afternoon), and 5:30pm-8:00pm (evening). In making this decision, we attempted to balance the likelihood of obscuring important temporal patterns in the secondary proximal outcomes with the degree of assessment burden, cognitive overload, potential reactance, and risk of premature treatment dropout posed by too frequent EMAs (Nahum-Shani et al., 2015; Nahum-Shani et al., 2014; Nahum-Shani et al., 2018). We also considered participant availability and states of receptivity, despite the fact that we may miss important opportunities for support by excluding night-time decision points (Klasnja et al., 2015; Nahum-Shani et al., 2015; Nahum-Shani et al., 2014; Nahum-Shani et al., 2018). The use of semi-random EMA prompts across each day will allow us to examine the degree to which the timing of intervention delivery influences intervention efficacy and engagement. Moreover, an evaluation of the frequency and timing of the decision points in the acceptability evaluations will inform the limited information we have in relation to how our proximal outcomes change over time.

### Intervention Options

At any of our given decision points, intervention options are the range of potential treatments that may be employed based on our tailoring variables and decision rules (see below) (Carpenter et al., 2020; Goldstein et al., 2017; Nahum-Shani et al., 2015; Nahum-Shani et al., 2014; Nahum-Shani et al., 2018). These can include different types of support (e.g., psychoeducation, feedback, reminders, tips, motivational messages, self-monitoring, goal-setting, planning behaviour, glanceable displays, coping skills training), support delivery modes (e.g., provision or availability of support), amount of support (e.g., dose or intensity), or support delivery media (e.g., phone calls, text messages) (Bakker et al., 2016; Goldstein et al., 2017; Heron and Smyth, 2010; Kim et al., 2019; Klasnja & Pratt, 2012; Nahum-Shani et al., 2014; Nahum-Shani et al., 2018). These intervention options, which should be designed for just-in-time delivery (i.e., precisely when people are in states of vulnerability or opportunity), are sometimes referred to as *EMIs* (Heron & Smyth, 2010; Nahum-Shani et al., 2014, 2018). These intervention options, which often target proximal outcomes, should be theoretically- and empirically-driven (Nahum-Shani et al., 2015; Nahum-Shani et al., 2014; Nahum-Shani et al., 2018).

The intervention options in *GamblingLess: In-The-Moment* were designed to target the cognitive processes which signal a state of cognitive vulnerability (cravings, lowered self-efficacy, and endorsement of positive outcome expectancies; secondary proximal outcomes) that increase the probability of a subsequent gambling episode (primary proximal outcome). The JITAI comprises 53 activities spanning three separate intervention modules: (1) *Curbing Cravings* (comprising ten craving management activities); (2) *Tackling Triggers* (comprising 25 activities to enhance self-efficacy in five high-risk situations: financial pressures, unpleasant emotions, social pressure to gamble, testing control over gambling, and conflict with others); and (3) *Exploring Expectancies* (comprising 18 activities to reduce positive outcome expectancies organised into three groups: excitement, escape, and money). Most intervention activities take less than five minutes to complete, consistent with the *GamblingLess: Curb Your Urge* pilot JITAI (Hawker et al., 2021; Merkouris et al., 2020). The relapse prevention model informed the development of intervention options (Larimer et al., 1999; Marlatt & Gordon, 1985; Witkiewitz & Marlatt, 2004), as well as acceptability feedback from the *GamblingLess* research program (Dowling et al., 2018, 2021; Hawker et al., 2021; Humphrey et al., 2020; Humphrey et al., 2022; Merkouris et al., 2020; Merkouris et al., 2017; Rodda et al., 2019). Hence, the strategies are primarily cognitive and behavioural strategies that focus on the immediate determinants of relapse, but include third wave approaches, including mindfulness-based and acceptance-based strategies (Larimer et al., 1999; Marlatt & Gordon, 1985; Marlatt & Witkiewitz, 2005; Witkiewitz & Marlatt, 2004). Cognitive-behavioural treatments are considered to be the gold standard intervention for gambling problems (Cowlishaw et al., 2012; Gooding & Tarrier, 2009; Goslar et al., 2017), with an emerging literature supporting the efficacy of mindfulness-based interventions (de Lisle et al., 2012; Maynard et al., 2018).

Consistent with the HAPA model (Schwarzer & Luszczynska, 2008), the intervention options for *Gambling Habit Hacker* were developed to target the cognitive and behavioural processes which signal states of goal vulnerability (low strength of intention, low goal self-efficacy, low urge self-efficacy, and high-risk situations; secondary proximal outcomes) for spending more than intended (primary proximal outcome). Prior research has identified multiple categories of self-enactable strategies gamblers use to adhere to their gambling limits (Hing et al., 2019; Rodda, K. L. Bagot et al., 2018; Rodda et al., 2019a, b), but that several factors, such as a failure to select fit-for-purpose strategies, an inability to sustain strategy use, shifting priorities, and using conflicting strategies, can influence strategy success (Rodda et al., 2017). Goal setting, action planning, coping planning, and self-monitoring were therefore selected as the intervention components for *Gambling Habit Hacker* to bridge the gap between intention and behaviour. This JITAI comprises 120 individual strategies (e.g., eat healthy) across 25 higher order strategy groups (e.g., support good health), which were further organised into 10 higher order behaviour change categories (avoidance, financial management, maintaining momentum, managing emotions, rewards, substitution activities, social support, staying in control while gambling, stress management, and urge management) to facilitate comparison with the broader evidence base (Michie et al., 2013; Rodda et al., 2018a, c, d).

In the action planning stage, individuals are prompted to select a tailored strategy group based on their responses to the tailoring variables, followed by a relevant strategy accompanied by implementation information drawn from lived experience research and prompts for personalising each specific strategy. Individuals are then prompted to record a personally tailored-action plan in an open text field. In the coping planning component, individuals are prompted to identify the main proximal barrier to the successful implementation of their action plan (thoughts, emotions, motivation, situation, self-belief, financial, and social), describe the details of the barrier that was selected in an open-text box, and record a detailed plan for this implementation barrier (Armitage, 2009). Finally, participants are encouraged to participate in commitment and self-efficacy activities focused on strength of character and mental rehearsal of the plan (Hamilton et al., 2019; Knäuper et al., 2009). We undertook extensive work to adapt all behaviour change strategies for in-the-moment delivery. For example, individuals selecting self-exclusion were prompted to engage in the next step required to implement this strategy (e.g., download the application form). Similarly, coping planning, which is usually undertaken ahead of time (Sniehotta et al., 2005), is prompted in real time by requesting individuals to consider immediate action to address the identified barrier. The intervention activities across the action and coping planning components take between 5 and 10 min to complete.

Importantly, both apps include a *Get More Support* feature, which enables click-to-call and click-to-email functions to helpline and web-based specialist gambling services. These direct linkages into other gambling treatment services allow individuals to escalate the type of support they wish to receive, which includes immediate crisis support (Bakker et al., 2016).

One of the biggest challenges when developing mHealth interventions is engagement with content and client attrition. Although mHealth interventions increase accessibility, they are characterised by high dropout levels and ‘non-usage attrition’ (unsustained engagement) (Attwood et al., 2017; Milward et al., 2018; Yardley et al., 2016). Intervention engagement and intervention fatigue, which fluctuate over time, affect intervention adherence, retention, and effectiveness (Carpenter et al., 2020; Kreyenbuhl et al., 2009; Milward et al., 2018; Nahum-Shani et al., 2018). Receptivity is therefore emphasised in JITAI designs (Nahum-Shani et al., 2015; Nahum-Shani et al., 2018). It is argued that the provision of support when individuals are not receptive is unhelpful and may even be deleterious by exacerbating intervention engagement and fatigue (Nahum-Shani et al., 2018).

To increase user engagement and minimise intervention fatigue, we aimed to create simple, aesthetically pleasing designs, and varied the way in which content was delivered in terms of its presentation, form, and timing. For example, rather than repeatedly delivering the same intervention content, we encouraged autonomy by incorporating users’ intervention option preferences, whereby they drew from a menu of relevant intervention activities. Moreover, intervention options are intuitive and easy to navigate, with optimal challenge and interest levels. Text was written in non-judgemental, inclusive, simple, and hopeful language and we considered the literacy of intended users in determining sentence and paragraph length. In *GamblingLess: In-The-Moment*, we incorporated intervention options that are interactive and gamified across multiple media platforms (video, audio, quizzes, personalised feedback, multiple-choice items, and open-ended items) and included a *Pick For Me* feature on each module menu, whereby individuals could allow the app to randomly select an intervention activity from the menu. We also repeatedly delivered brief static psychoeducational messages via a *Did You Know?* feature to reduce text. In *Gambling Habit Hacker*, we provided space to develop customised plans and included quotes representing the lived experience of gamblers to enhance relatedness (Deci & Ryan, 2008; Ntoumanis et al., 2021; Sheeran et al., 2020).

It was also important to consider the ethics of providing interventions in real-life settings in terms of confidentiality, privacy, safety and general welfare of the individual. For this reason, both apps include a “provide nothing” option in the form of a “snooze” function, for use in situations in which the individual does not require support or is unreceptive (e.g., ignores the EMA prompt), or when providing support may be inconvenient, unethical, or unsafe (Klasnja et al., 2015; Nahum-Shani et al., 2015; Nahum-Shani et al., 2014; Nahum-Shani et al., 2018). Individuals can complete an EMA within a two-hour window after the initial notification to preserve the momentary nature of the treatment while accommodating the potential for their possible unavailability at the initial notification time (Goldstein et al., 2017; Klasnja et al., 2015). We will therefore estimate the influence of the intervention on the proximal outcomes among people who are available for treatment at any given decision point (Klasnja et al., 2015). Moreover, for *GamblingLess: In-The-Moment*, we included an indication of the modality of each activity on each menu (e.g., text, video, interactive, audio, or text and image) so individuals can make an informed decision regarding appropriate intervention activities in their current situation. For *Gambling Habit Hacker*, intervention content was presented for a range of contexts and situations, including preparing for a gambling session through to gambling in a venue.

## Formulating the Adaptation Strategy

### Tailoring Variables

Tailoring variables are used to make decisions about when and how to intervene (Carpenter et al., 2020; Collins et al., 2004; Nahum-Shani et al., 2015; Nahum-Shani et al., 2014; Nahum-Shani et al., 2018). Tailoring variables can be obtained using passive assessments, active assessments, or both (Kim et al., 2019; Nahum-Shani et al., 2014, 2018; Wang & Miller, 2020). Passive assessments, which are low-burden because they require no or minimal user engagement, use sensor-equipped smartphones and wearable devices to collect automated data (e.g., physical activity, temperature, location, light, sound, sleep, blood pressure, heart rate, respiration rate, social interactions, camera images) to make inferences about internal states and contexts (Hekler et al., 2013; Klasnja & Pratt, 2012; Riley et al., 2015). In JITAIs, active assessments, which are higher burden because they require user engagement and compliance, are also known as EMAs (Nahum-Shani et al., 2018). An EMA design is an event-level prospective methodology that involves the repeated measurement of self-reported symptoms, emotions, behaviour, thoughts, and context in real-time and in natural environments, usually via smartphones (Shiffman, 2009; Shiffman et al., 2008; Stone & Shiffman, 1994). Although recent evidence suggests that there is no significant difference in JITAI outcomes when active and passive assessments are employed (Wang & Miller, 2020), passive data collection does not comprehensively and accurately evaluate internal states, such as mood, craving, and cognitions (Carpenter et al., 2020; Kim et al., 2019; Stone & Shiffman, 1994). EMA has the added advantage over passive assessments of facilitating accurate self-monitoring, which in turn can lead to increases in emotional self-awareness and regulation (Bakker et al., 2016; Heron and Smyth, 2010; Klasnja et al., 2015; Walton et al., 2018).

For these reasons, the tailoring variables for *GamblingLess: In-The-Moment* and *Gambling Habit Hacker* are assessed using an in-app EMA protocol employing time-based sampling (i.e., using semi-random prompts for people to input their internal states and situational contexts) that incorporated event-based sampling (e.g., collecting gambling episode and expenditure data). Because tailoring variables with low validity can produce high rates of false positives, the tailoring variables employed in both apps were derived from validated scales or previous EMA and EMI research (Collins et al., 2004; Goldstein et al., 2017; Nahum-Shani et al., 2014; Nahum-Shani et al., 2018).

For *GamblingLess: In-The-Moment*, we selected three tailoring variables (craving intensity, self-efficacy, and positive outcome expectancies) that signal an emerging cognitively vulnerable state. Each EMA therefore included single items assessing each of these constructs, which were scored on various 5-point response scales (from 0 to 4, where higher scores indicate higher vulnerability) (Dowling et al., 2022). EMAs also included items assessing other momentary internal states and situational contexts that will be employed to explore the conditions under which the JITAI is more or less effective, including psychological distress, readiness to change, subjective alcohol intoxication, impulsivity, social context, financial gambling availability, and location gambling availability (Dowling et al., 2022).

*Gambling Habit Hacker* also employed tailoring variables based on the app’s proximal outcomes that signal the emergence of goal vulnerability (i.e., low strength of intention, low goal self-efficacy, low urge self-efficacy, and high-risk situations) for subsequent non-adherence to gambling expenditure limits. These tailoring variables target both the motivational (strength of intention for goal adherence) and volitional (ability to implement and maintain actions that facilitate goal adherence) phases of the HAPA model (Schwarzer, 2008). The 18-item EMA protocol for this app comprised single items measuring strength of intention, goal self-efficacy, and urge self-efficacy and 15 items measuring high-risk situations (including negative reinforcement, positive reinforcement, alcohol consumption, and gambling proximity), each scored on various 5-point response scales (Rodda et al., 2022) (from 1 to 5, whereby higher scores indicate lower goal vulnerability for strength of intention and goal self-efficacy; and higher goal vulnerability for urge self-efficacy and high-risk situations).

The type, timing, and amount of support can be tailored to individual needs in a JITAI (Heron & Smyth, 2010; Nahum-Shani et al., 2015; Nahum-Shani et al., 2014; Nahum-Shani et al., 2018; Wang and Miller, 2020). In both apps, we individualise the *type* of treatment by using the EMA tailoring variables to determine which intervention module (*GamblingLess: In-The-Moment)* or strategy group *(Gambling Habit Hacker)* an individual will receive. In both apps, we individualise the *timing* of treatment by delivering interventions at times when individuals are particularly in need of support (Heron & Smyth, 2010) but not when they do not require support or are not in a state of receptivity. Finally, in *GamblingLess: In-The-Moment*, we tailor the *amount* or dosage of support to individual needs using an intervention loop, which continues until the individual no longer requires support (based on responses to specific post-intervention activity EMA items) or closes the app. Should data allow, we may use these data explore the degree to which each index of cognitive vulnerability improves immediately after the delivery of an intervention activity.

### Decision Rules

Pre-defined decision rules operationalise the adaptation in a JITAI by specifying which intervention option is offered, to which people, and under which contexts (Carpenter et al., 2020; Nahum-Shani et al., 2015; Nahum-Shani et al., 2014; Nahum-Shani et al., 2018). Because the tailoring variables and intervention options are linked in a systematic way in decision rules, each decision point is associated with a decision rule (Nahum-Shani et al., 2014, 2018). Decision rules include the values (which can be levels, thresholds, or ranges) of each tailoring variable that indicate which intervention option should be offered to each individual (Nahum-Shani et al., 2014, 2018). Most available JITAIs employ decision rules that are expressed as a series of conditional statements (e.g., if craving > [threshold], then JITAI recommends delivery of a craving management intervention) (Carpenter et al., 2020; Nahum-Shani et al., 2015; Nahum-Shani et al., 2014; Nahum-Shani et al., 2018).

In *GamblingLess: In-The-Moment*, eligibility for an intervention is determined based on the individual’s momentary level of craving intensity (tailoring variable 1), self-efficacy (tailoring variable 2), and positive outcome expectancies (tailoring variable 3). At each decision point, individuals who fail to reach the cut-off point on any tailoring variable (i.e., score zero) are not eligible for an intervention but are sent an encouraging message. In contrast, individuals who exceed the cut-off point (i.e., score one or more) on one or more of these tailoring variables are eligible to be delivered a tailored intervention (i.e., Curbing Craving, Tackling Triggers, or Exploring Expectancies). Given that the reformulated relapse prevention model does not postulate that some factors have more influence in determining relapse than other factors (Witkiewitz & Marlatt, 2004), individuals who are eligible for more than one intervention module will be randomly allocated to one of those modules. After an intervention activity is completed, the intervention loop is potentially triggered by an individual’s response to a post-intervention EMA item, which is subject to the same decision rules. Individuals who exceed the cut-off point are presented with a personalised feedback message and returned to the relevant intervention dashboard. Because there was insufficient empirical evidence to identify the cut-off point of each tailoring variable, we do not know the level of each tailoring variable at which the delivery of each intervention module is likely to be beneficial versus unnecessary. Although the proposed trial design will not allow for an evaluation of the causal effect of providing recommendations based on different levels of each tailoring variable (Nahum-Shani et al., 2015), we have deliberately set low eligibility thresholds so we can potentially optimise the intervention by exploring the influence of different cut-points on treatment outcomes.

In *Gambling Habit Hacker*, intervention eligibility is determined based on the individual’s momentary level of strength of intention (tailoring variable 1), goal self-efficacy (tailoring variable 2), urge self-efficacy (tailoring variable 3), and high-risk situations (tailoring variable 4 comprising 15 situations). Cut-points vary across these tailoring variables: score of 3 or less for strength of intention and goal self-efficacy, score of 3 or more for urge self-efficacy, and score of 2 or more on high-risk situations. At each decision point, individuals who exceed the cut-off point on each tailoring variable are eligible for an intervention, whereby they are delivered between 12 and 25 of the 25 available strategy groups. A pre-determined hierarchy determines which strategies are delivered in the event that individuals exceeded the cut-points on multiple tailoring variables (see Rodda et al., 2022). In this hierarchy, threats to adhering to gambling expenditure limits were ordered from most to least: gambling proximity (engagement in planned/unplanned gambling or planned gambling day), reduced urge self-efficacy, being in a high-risk situation, reduced strength of intention, and reduced goal self-efficacy. In contrast, individuals who fail to reach the cut-off point on any tailoring variable are not eligible for an intervention but are sent an encouraging message.

## The Application of Micro-Randomised Trials

Collins and colleagues (Collins et al., 2009; Collins et al., 2005) have proposed that factorial trial designs form part of the Multiphase Optimization Strategy (MOST), which is a framework for engineering effective multi-component behavioural interventions. While traditional factorial designs can be employed to explore the influence of each intervention component and important interaction effects, they are unable to determine the conditions under which each intervention component is most effective (Klasnja et al., 2015). MRTs, which are a type of sequential factorial design in which each person is randomly allocated to intervention options at each decision point across a pre-specified period of time, overcome these limitations (Collins et al., 2009; Collins et al., 2005). Moreover, MRTs are a highly efficient trial design because the within-subject comparisons in which participants act as their own control group require smaller sample sizes than traditional full factorial designs (Klasnja et al., 2015). Although MRTs are an emerging experimental design, there are several illustrations of their use in addiction science (Carpenter et al., 2020). MRTs are specifically designed to enable the optimisation of JITAIs, which involves deciding the ways in which a JITAI should be adjusted to make it more effective, efficient, and scalable (Collins & Kugler, 2018; Collins et al., 2005; Klasnja et al., 2015). Optimisation is particularly important in JITAI design, given the potential burden and disengagement resulting from these interventions (Collins & Kugler, 2018; Collins et al., 2005; Walton et al., 2018). Optimisation involves investigating the effectiveness of each component (decision point, intervention option, tailoring variable, and decision rules) and how well these components operate together (Collins & Kugler, 2018; Collins et al., 2005). MRTs provide empirical data for optimising JITIAIs by examining how and under what conditions we should deliver intervention options to enhance their effectiveness (Carpenter et al., 2020).

We will employ this trial design to inform the optimisation of both of our JITAIs. We made the decision to conduct MRTs for these JITAIs because there is insufficient empirical and theoretical evidence to fully construct the decision rules that precisely specify when specific intervention components can be delivered to maximise their effects (i.e., to identify the decision points, tailoring variables, and intervention options that would form the most effective intervention) (Carpenter et al., 2020; Klasnja et al., 2015). In both MRTs, participants will be randomly allocated to a tailored intervention condition or a no intervention control condition at each decision point across 28-days. With three decision points per day, each participant can be randomised up to 84 times across each MRT. In the micro-randomisation protocol for *GamblingLess: In-The-Moment*, eligible participants will have a 75% chance of being micro-randomised into the tailored intervention condition and a 25% chance of being micro-randomised into the no intervention control condition. Participants who are eligible for two intervention modules will have a 37.5% chance of receiving either intervention module; and participants who are eligible for all three intervention modules will have a 25% chance of receiving any of the intervention modules. In this trial, participants who are micro-randomised to the no intervention control condition will be delivered a brief tailored message but no intervention activities. In the micro-randomisation protocol for *Gambling Habit Hacker*, eligible participants will have a 50% chance of being micro-randomised into the tailored intervention and no intervention control condition. In this trial, participants who are micro-randomised to the no intervention control condition will be presented with the names of the 25 self-enactable strategies but no implementation guidance. We selected a higher ratio of intervention allocation for *GamblingLess: In-The-Moment* because we wanted, should data allow, to explore which of the three intervention options was most beneficial.

MRTs are capable of answering four critical scientific questions that may be helpful when attempting to optimise a JITAI (Carpenter et al., 2020; Klasnja et al., 2015; Walton et al., 2018). Specifically, they can be used to inform decisions about: (1) whether or not an intervention option should be included by exploring causal proximal effects of randomised specific intervention components; (2) which intervention options should be included by comparing the proximal influence of multiple randomised specific intervention options; (3) under what conditions individuals should be interrupted to provide an intervention option, or one type of intervention option over another, by examining how the proximal influence of intervention options vary depending on the timing of support, individual internal states, and situational contexts; and (4) when an intervention option should be delivered, or how different intervention options should be sequenced, by exploring how the proximal influence of intervention options change over the duration of the treatment (Carpenter et al., 2020; Klasnja et al., 2015; Nahum-Shani et al., 2014; Nahum-Shani et al., 2018; Walton et al., 2018).

The primary aim of both MRTs is to explore the degree to which it is worthwhile to deliver a tailored intervention option at a time of cognitive vulnerability (*GamblingLess: In-The-Moment*) and goal vulnerability (*Gambling Habit Hacker*). Compared with the delivery of no intervention, the MRTs aim to explore whether the delivery of a tailored intevention either reduces the probability of a subsequent gambling episode and improves craving intensity, self-efficacy, and positive outcome expectancies (*GamblingLess: In-The-Moment)* or increases adherence to subsequent gambling expenditure limits and improves strength of intention, goal self-efficacy, and urge self-efficacy (*Gambling Habit Hacker*).

Should data allow, secondary exploratory research questions for the *GamblingLess: In-The-Moment* MRT include: (1) Is the delivery of one intervention option (targeting cravings, self-efficacy, or positive outcome expectancies) more likely to reduce the probability of a subsequent gambling episode than the other intervention options?; (2) How do time-variant (EMA) factors (time of day, time of week, craving intensity, self-efficacy, positive outcome expectancies, psychological distress, impulsivity, subjective alcohol intoxication, readiness to change, gambling availability (financial), gambling availability (location), and social context) and time-invariant (pre-intervention) factors (gambling symptom severity, gambling frequency, gambling expenditure, gender, and age) influence the intervention effect on the probability of a subsequent gambling episode?; and (3) How does the effect of a tailored intervention on the probability of a subsequent gambling episode change over the course of the 28-day MRT? We may also explore the degree to which, compared to the delivery of no intervention, the delivery of: (a) a craving intervention reduces subsequent craving intensity; (b) a self-efficacy intervention increases subsequent self-efficacy; and (c) a positive outcome expectancy intervention decreases subsequent positive outcome expectancies. Similarly, should data allow, secondary exploratory research questions for the *Gambling Habit Hacker* MRT include: (1) How do time-variant (EMA) factors (strength of intention, goal self-efficacy, urge self-efficacy or being in a positive or negative high-risk situation, alcohol or drug consumption, and gambling proximity) and time-invariant (pre-intervention) factors (age, gender, volitional phase, gambling symptom severity, gambling expenditure, and planning propensity) influence the intervention effect on subsequent adherence to gambling expenditure limits?; and (2) How does the effect of the tailored intervention on subsequent adherence to gambling expenditure limits change over the course of the 28-day MRT?

## Challenges and Future Directions

We encountered several logistical and methodological challenges when developing these JITAIs, particularly in relation to applying an MRT design to their evaluation. Some of these challenges have been identified by other researchers (Goldstein et al., 2017). A selection of these issues, along with future research considerations, are discussed below.

### Evaluation Considerations

Evaluations of JITAIs, particularly using MRTs, are characterised by a lack of longer-term follow-up evaluations. This is particularly problematic for these interventions, given that part of their rationale is that they encourage the use of skills in everyday life, thereby increasing the probability of long-lasting behaviour change (Heron & Smyth, 2010; Krebs et al., 2010; Wang & Miller, 2020). We therefore decided to supplement the MRTs with six-month within group follow-up evaluations of both apps to examine within-group change over a longer period of time, as well as to identify the factors that predict these longer-term treatment outcomes. These evaluations will also conduct supplementary analyses of the clinical significance of the JITAIs in terms of meaningful changes in people’s lives by reporting effect sizes and classifying the status of each participant as recovered, improved, unchanged, or deteriorated on outcome variables according to reliable change indices (Jacobson & Truax, 1991).

There is also widespread consensus that successful JITAI designs requires a user-centred and iterative approach to development, whereby both mixed methods and in-depth qualitative methods can be used to refine the intervention to meet the needs of the users (Heron & Smyth, 2010; Yardley et al., 2016). Consistent with these recommendations, *GamblingLess: In-The-Moment* was built on the user, acceptability, and pilot data provided by people with lived experience for *GamblingLess: Curb Your Urge* (Hawker et al., 2021; Merkouris et al., 2020). Similarly, the perspectives of people with lived experience of gambling problems are represented in the development of *Gambling Habit Hacker*, whereby the implementation support was sourced from lived experience accounts from more than 2000 gamblers across counselling transcripts, online forums, in-venue surveys, and community-based qualitative and quantitative surveys (Bagot et al., 2021; Rodda et al., 2017, 2018a, c, d, 2019b; Rodda, Hing, Rodda et al., 2018a, c, d). Prior to each evaluation, we also subjected both apps to user testing with gambling stakeholders, with feedback indicating that they are acceptable gambling interventions (Dowling et al., 2022; Rodda et al., 2022). Moreover, we will explore the acceptability of both JITAIs with trial participants using both quantitative and qualitative methods, including surveys at post-intervention, indices of app use and engagement, and semi-structured interviews.

### On-demand Intervention Content

In the organising framework provided by Nahum-Shani et al. (2018), JITAIs are defined as “push” intervention approaches, in which it is assumed that individuals are often unaware of the emergence of states of vulnerability and/or opportunity or are unmotivated to access the support they require to manage these states (Nahum-Shani et al., 2015; Nahum-Shani et al., 2018). This approach contrasts with “pull” approaches to intervention delivery that require individuals to be able to recognise states of vulnerability and be sufficiently motivated to initiate access to the support they need (Klasnja et al., 2015; Walton et al., 2018).

In designing these JITAIs, we were aware that some individuals may have higher emotional self-awareness than others and that the EMA protocol could enhance the recognition of these states over time (Bakker et al., 2016; Heron and Smyth, 2010; Klasnja et al., 2015; Walton et al., 2018). From clinical and ethical perspectives, we wanted to encourage individuals to practice the coping skills delivered by the apps in their everyday lives when they recognise states of vulnerability and/or opportunity and are motivated to access support, with a view to enhancing the generalisation of learned skills to new settings and maintain therapeutic gains (Bakker et al., 2016; Heron and Smyth, 2010; Klasnja et al., 2015; Walton et al., 2018). Although they exclude interventions that solely rely on individuals initiating and selecting from available support options, Nahum-Shani et al. (2018) acknowledge that the addition of some “participant-determined features” to a JITAI may provide some advantages. Adding such features may accommodate conditions in which individuals are in the best position to know when support is required and what type of support would be helpful, facilitate autonomy through agency and control, and reduce disruption as long as they do not access support when they are unreceptive (Fukuoka et al., 2012; Nahum-Shani et al., 2018). Nahum-Shani et al. (2018) suggest, however, that further research is required to evaluate how to best add these features to a JITAI to ensure that planned and externally-initiated support is balanced with personal volition.

In designing both JITAIs, we considered allowing individuals to initiate decision points, in addition to the three protocol-driven decision points each day. Participant-initiated decision points are those in which the user requests support or when the user accesses the intervention content on-demand (Nahum-Shani et al., 2014). Participant-initiated decision points have been employed in previous JITAIs (Ben-Zeev et al., 2014; Businelle et al., 2016; Franklin et al., 2008; Free et al., 2011; Gustafson et al., 2014). In this model, however, the micro-randomisation protocol would not be applied to participant-initiated decision points. Moreover, the on-demand intervention content would be untailored and easier to access than that delivered via the more time-intensive EMAs, which would likely impact on the feasibility of the MRTs. We also considered allowing participants to access tailored intervention content via participant-initiated EMAs (Nahum-Shani et al., 2014), in addition to the three protocol-driven EMAs each day, and subjecting both types of EMAs to the same MRT protocol. In this design, EMAs can be initiated by the participant in addition to the automated system (Businelle et al., 2016; Free et al., 2011). This design, however, may also influence the feasibility of the MRTs as eligible participants who are randomly assigned to the no intervention condition at any given decision point can immediately access the intervention content via the on-demand feature.

Klasjna et al. (2015) argue that MRTs are only appropriate for evaluating push interventions and are not appropriate for evaluating pull intervention components. In contrast, Walton et al. (2018) argues that MRTs can be used to evaluate both push and pull interventions, but focus their discussion on push interventions. On balance, we decided to maintain the integrity of the MRT evaluations by excluding access to intervention content on-demand during the MRT period, then allowing access to this content via participant-initiated EMAs during the six-month follow-up periods when participants recognise they are in a state of vulnerability and are motivated to access support. We will explore whether trial participants prefer a more traditional pull approach or the addition of participant-determined features for on-demand intervention content in the acceptability evaluations for both apps.

### Intervention Optimisation

The optimisation of these JITAIs may involve us removing less effective components, and determining when and in what contexts different treatments should be offered to maximise efficiency and minimise burden ((Klasnja et al., 2015; Walton et al., 2018). Moreover, for both apps, we will be able to refine the decision rules by identifying appropriate timing of the intervention and cut-points for each tailoring variable. An RCT to evaluate the intervention compared to other interventions is appropriate only when there is sufficient empirical support for the optimal delivery of the intervention components (Carpenter et al., 2020; Collins et al., 2009; Collins et al., 2005; Walton et al., 2018). Further, while conditional statements are appropriate when there are relatively few statements, the complexity of the model underpinning a JITAI expands exponentially as a result of adding additional contextual considerations or intervention tailoring options (Goldstein et al., 2017). A more rigorous method of codifying behaviour is to develop mathematical models of the decision process using machine learning methods (Goldstein et al., 2017). Machine learning, which is a subfield of artificial intelligence, can produce highly accurate predictive models from large datasets and automatically adapt to new data in real time (Goldstein et al., 2017; Gustafson et al., 2014; Nahum-Shani et al., 2014; Wang and Miller, 2020). Moreover, because excessively broad conditional statements that apply to all users could result in inappropriate intervention delivery to any specific individual (Goldstein et al., 2017), machine learning approaches could be used to continually re-adapt decision rules for each individual over time (Goldstein et al., 2017; Kim et al., 2019; Nahum-Shani et al., 2018; Riley et al., 2015).

### Combining Active and Passive Assessments

Although EMA procedures are the gold-standard methodology for assessing dynamic internal states (Carpenter et al., 2020; Kim et al., 2019; Stone & Shiffman, 1994), they require high participant engagement and compliance, are associated with some degree of recall and reporting bias, and possibly involve assessment reactivity (Goldstein et al., 2017; Kim et al., 2019; Nahum-Shani et al., 2014; Nahum-Shani et al., 2018). In future versions of both JITAIs, it may be possible to augment EMA data with some tailoring variables obtained from passive assessments, which could reduce the burden on users, provide more contextual information, and enhance user awareness of behaviour (Goldstein et al., 2017; Kim et al., 2019). For example, data from sensors or other technologies to detect location (proximity to a land-based gaming venue using GPS), social interactions (ambient noise detection), increased heart rate (EEG), or states of unavailability or receptivity (e.g., driving, exercising, working, or sleeping), could be employed to mark states of heightened vulnerability to gambling episodes and expenditure (Goldstein et al., 2017; Gustafson et al., 2014; Kim et al., 2019; Nahum-Shani et al., 2014; Wang and Miller, 2020).

### Human Facilitation

Studies often provide JITAIs as only one intervention component in a treatment protocol (Heron & Smyth, 2010), with evidence that guided m-Health interventions are generally associated with superior treatment outcomes to unguided interventions (Baumeister et al., 2014). Future iterations of these interventions could add personal coaches and assistants via digital avatars (Fogg, 2007) or the involvement of coaches, guides or therapists to maintain engagement, motivation, and adherence to intervention requirements (Gustafson et al., 2014; Klasnja & Pratt, 2012; Mohr et al., 2011; Yardley et al., 2016). For example, remote coaching (in which healthcare providers review the data and work with individuals to support them in managing their conditions) and remote symptom monitoring (in which healthcare providers are alerted if concerning symptoms develop) can inform healthcare providers about the individual’s condition and enhance the care interactions between providers and patients (Klasnja & Pratt, 2012). We could also use these JITAIs to supplement face-to-face or mobile psychological and behavioural therapies or deliver “booster” treatments following these interventions to consolidate behaviour change (Heron & Smyth, 2010). Although the evidence for social support in mHealth interventions remains unclear (Milward et al., 2018), the addition of social support among individuals who share the same condition and goals (peer-to-peer influence) and peers who have succeeded in achieving similar goals (peer modelling) could facilitate supportive social interactions and increase feelings of relatedness and connectedness (Heron & Smyth, 2010; Klasnja & Pratt, 2012; Yardley et al., 2016). The degree to which trial participants prefer the involvement of clinicians, guides, coaches, peers, or digital avatars will be explored in the acceptability evaluations for both apps. Given that unguided interventions can be effectively delivered at lower cost (Baumeister et al., 2014; Yardley et al., 2016), however, future research is necessary to establish for whom and when providing human support adds value.

### Cost-effectiveness Evaluations

JITAIs are promoted as cost-effective solutions to health behaviour change due to their potential to improve the efficacy of treatment and reduce overall treatment duration (Heron & Smyth, 2010). There are, however, cost considerations such as the price of purchasing treatment software and hardware, as well as the time needed to set up and implement them (Heron & Smyth, 2010). Although there are some indications of the costs of JITAI treatment (Przeworski & Newman, 2004), there are few cost-effectiveness studies designed to facilitate decisions about how healthcare expenditure is best spent (Agras et al., 1990). Future research employing cost evaluation analyses that weigh up the relative costs and outcomes of these two JITAIs with other interventions are therefore required to inform decisions about resource allocation (Heron & Smyth, 2010).

### Transdiagnostic JITAIs

Finally, there is scope for redeveloping both apps to deliver transdiagnostic interventions across the addictions. Transdiagnostic approaches represent a paradigm shift from the study of discrete diagnostic categories to conceptualising psychopathology as comprising higher-order underlying dimensions which are shared across multiple, apparently distinct, conditions within individuals. The recent Component Model of Addiction Treatment suggests that targeting enduring, but modifiable, vulnerabilities that are common to all behavioural and substance use addictions could serve to improve the efficacy, efficiency, and cost-effectiveness of treatment (Kim & Hodgins, 2018). The empirical support for the highly influential relapse prevention model across addictive behaviours indicates that *GamblingLess: In-The-Moment* could be redeveloped to target the cognitive processes that precipitate episodes of behaviour and use across all behavioural and substance use addictions. Similarly, *Gambling Habit Hacker* forms part of a suite of implementation planning interventions across the addictions, including gaming, alcohol and sugar (Brittain et al., 2021; Park et al., 2020; Rodda et al., 2018c), suggesting that it could be redeveloped as a transdiagnostic intervention to help people with a range of addictions set their behavioural intentions and support adherence to their limits in real-time and real-world settings.

## Conclusion

JITAIs are emerging “push” mHealth interventions that adapt the provision of the type, amount, or timing of support to an individual’s dynamic needs. JITAIs, which have demonstrated effectiveness across a range of health domains, are particularly suited to the treatment of addictions. We attempted to redress our current gap in service provision by developing two JITAIs, *GamblingLess: In-The-Moment* and *Gambling Habit Hacker*. We applied an organising framework (Nahum-Shani et al., 2015; Nahum-Shani et al., 2014; Nahum-Shani et al., 2018) to the development of these two JITAIs. These two interventions were designed to meet the needs of both higher- and lower-risk gamblers to expand access to theoretically-informed and evidence-based treatments to individuals across the continuum of gambling risk who may be experiencing harm from their gambling.

Consistent with their respective theoretical underpinnings, we constructed different decision rules for each of these JITAIs. *GamblingLess: In-The-Moment* aimed to provide the right type and amount of support required at times of cognitive vulnerability characterised by high craving intensity, lowered self-efficacy, and positive outcome expectancies to reduce the likelihood of a subsequent gambling episode, with a view to reducing gambling symptom severity in the longer-term. In contrast, *Gambling Habit Hacker* aimed to provide the type of support required at times of goal vulnerability characterised by low goal intention strength, low goal self-efficacy, low urge self-efficacy, and high-risk situations to enhance adherence to gambling expenditure limits, with a view to reducing gambling expenditure in the longer-term. Given insufficient theoretical and empirical evidence to fully construct their decision rules, we plan to evaluate these JITAIs with MRTs so that we can obtain the empirical data that is necessary for their optimisation. While JITAIs appear to be a promising intervention design in addiction science, we identified several key challenges and considerations for future research. We therefore described the decisions, methods, and tools we employed in the development of *GamblingLess: In-The-Moment* and *Gambling Habit Hacker*, as well as considerations of these challenges and future research directions, as they are likely to apply to future JITAIs that target other addictive behaviours.

## Data Availability

No data was generated or analysed in this manuscript.
